# Three-Dimensional Analysis of Morphological Adaptation and Wear in Restorations Performed Using the Stamp Technique with Different Viscosity Composite Resins

**DOI:** 10.3390/biomimetics11060420

**Published:** 2026-06-13

**Authors:** İlknur Akay Dede, Ayşenur Yazım, Cemile Kedici Alp

**Affiliations:** Department of Restorative Dentistry, Faculty of Dentistry, University of Gazi, 06490 Ankara, Turkey

**Keywords:** biomimetics, composite resins, tooth wear, dental materials, dental restoration

## Abstract

The stamp technique is a biomimetic approach that enables accurate reproduction of preoperative occlusal morphology in direct composite restorations; however, the rheological properties of restorative materials may influence both morphological adaptation and wear behavior. This in vitro study aimed to evaluate the morphological accuracy of different composite resins applied using the stamp technique and to quantify volumetric changes after toothbrushing simulation using three-dimensional analysis. Sixty standardized mandibular first molar model teeth were assigned to four groups (*n* = 15): Filtek Z250, Filtek One Bulk Fill, SonicFill 3, and G-ænial Universal Injectable. Digital scans were obtained at baseline, after restoration, and after brushing, and analyzed using OraCheck software to calculate volumetric gain (T0–T1) and volumetric loss (T1–T2). Significant differences were observed among groups for both outcomes (*p* < 0.001). G-ænial Universal Injectable showed the highest morphological accuracy but also the greatest wear, whereas SonicFill demonstrated lower morphological accuracy with superior wear resistance. No significant correlation was found across all groups; however, within each group, restorations with lower morphological accuracy tended to exhibit greater wear. These findings indicate that morphological accuracy and wear resistance are material-dependent and suggest that achieving a balance between accurate reproduction and long-term preservation of occlusal morphology remains a challenge.

## 1. Introduction

Biomimetic restorative dentistry focuses on preserving sound dental tissue, reproducing natural tooth anatomy, ensuring material compatibility with dental structures, and achieving a functional occlusal relationship [1]. Restorations that do not adequately reproduce the original tooth anatomy may cause occlusal imbalance and related functional problems [2]. Habitual intercuspation is the mandibular position reached during normal jaw closure and usually corresponds to maximum intercuspation in the absence of premature contacts [3]. Achieving anatomical conformity during restoration improves functional outcomes and boosts patient confidence and acceptance of the treatment [4]. Consequently, restoring occlusal morphology accurately in posterior teeth is clinically important, although achieving natural occlusal anatomy with direct composite restorations can be challenging and time-consuming, especially for clinicians with limited experience [5]. Moreover, free-hand reproduction of anatomical morphology with direct composite restorations may be particularly challenging in pediatric patients with limited cooperation. Planning and simulating the appropriate tooth morphology before treatment and accurately transferring it to the treated tooth may therefore improve clinical outcomes, suggesting that techniques such as the stamp technique can be clinically beneficial in such situations [6]. For this reason, the stamp technique has become increasingly used in composite restorations recently. This technique offers clinical advantages by reducing the need for occlusal adjustments, shortening finishing and polishing time, and preventing unnecessary composite use. This technique aims to replicate the natural tooth structure by transferring the existing occlusal morphology to the final restoration, thereby reducing the need for extensive occlusal adjustment and preserving the tooth anatomy [4,7,8]. Therefore, achieving and preserving the reproduced morphology is consistent with the fundamental principles of biomimetic restorative dentistry [9].

The stamp technique simplifies the restoration of occlusal anatomy, and the properties of the restorative material used also influence its effectiveness. In this context, material selection is important, as the viscosity of composite resins affects morphological adaptation. Clinicians increasingly prefer bulk-fill composites due to their simplified application, thanks to advances in composite restorative materials [10]. Bulk-fill resin-based composites represent a recent development in restorative materials and were introduced to allow for placement in thicker increments (approximately 4–5 mm), thereby reducing the time required for incremental layering and minimizing the risk of defects between layers. Their increased translucency and modified filler characteristics improve light transmission and depth of cure, which facilitates the bulk-filling approach [11]. SonicFill is a bulk-fill composite that can be polymerized in increments of up to 5 mm through sonic activation and combines properties of both flowable and packable composite resins. During placement, a dedicated handpiece applies sonic energy to temporarily reduce the viscosity of the material, facilitating adaptation to the cavity walls. Once the sonic activation stops, the material returns to a more viscous, sculptable consistency [12]. Furthermore, modifications in composite viscosity have led to the development of flowable resin-based composites, designed to improve adaptation to cavity walls and complex tooth morphologies. Flowable resin-based composites were developed by modifying the viscosity of conventional resin composites through increased resin content and reduced filler loading. These materials offer advantages such as improved adaptation to cavity irregularities, reduced air gaps, favorable esthetics, and biocompatibility. However, they may also exhibit limitations, including inferior mechanical properties, higher polymerization shrinkage, increased water sorption, and potentially reduced longevity [13]. Therefore, considerable efforts have been directed toward improving their mechanical performance, leading to the development of highly filled flowable composites with increased filler content to enhance wear resistance and long-term stability [14]. In the literature, these materials are commonly referred to as highly filled flowable composite resins, with filler contents typically ranging from approximately 61% to 71% by weight, enabling them to withstand higher occlusal loads [15].

The wear of teeth and restorative materials in the oral environment is a complex, multifactorial process. Among the factors contributing to composite degradation, toothbrushing is considered an important source of abrasion that occurs independently of occlusal loading. During brushing, toothbrush bristles and abrasive particles present in toothpastes generate mechanical stresses on the resin matrix, filler particles, and the filler-matrix interface, which may influence the wear resistance of restorative materials [16].

The composition and particle size of fillers directly affect surface hardness and wear behavior. Abrasive particles in toothpastes may cause physical degradation on restorative material surfaces during brushing [17]. In stress-free regions, one of the most common causes of material loss is brushing-induced abrasion. Therefore, the brushing-abrasion performance of restorative materials is essential for durability and esthetic stability [18]. Although obtaining accurate occlusal morphology at baseline using the stamp technique has clinical value, long-term preservation of this morphology depends on surface durability and wear resistance. Hence, initial morphological adaptation and post-brushing volumetric loss should be evaluated together. Surface morphology plays an important role in the long-term clinical performance of restorations [19]. Alterations in occlusal anatomy and surface topography may promote plaque accumulation, compromise oral hygiene, increase the risk of secondary caries, and negatively affect occlusal function. Furthermore, wear-related morphological changes may reduce restoration longevity and esthetic appearance over time [19,20]. Therefore, maintaining the original morphology achieved during restoration is clinically as important as obtaining accurate morphology at baseline.

Digital analysis methods are increasingly used to assess morphological changes reliably. Superimposing data sets obtained at different time points to perform three-dimensional (3D) deviation analysis enables new indications in dentistry [21]. These deviation analyses are used for evaluating volumetric changes such as tooth movement, gingival recession, and wear [22]. OraCheck 5.0.0 (Dentsply Sirona Deutschland GmbH, Bensheim, Germany) can be used to perform three-dimensional analysis of surface discrepancies and quantitative assessment of volumetric changes [23,24,25]. The ability of the software to detect minimal volumetric differences with high accuracy enables the identification of small morphological losses under clinical or in vitro conditions [26].

Although previous studies have investigated the stamp technique, composite wear behavior, and digital wear assessment separately, few studies have evaluated the relationship between the morphological accuracy achieved using the stamp technique and the subsequent wear behavior of restorative materials with different viscosity characteristics. Moreover, the influence of composite viscosity on both morphological accuracy and volumetric wear has not been comprehensively evaluated using three-dimensional digital analysis methods. Although obtaining accurate occlusal morphology at baseline using the stamp technique has clinical value, long-term preservation of this morphology depends on surface durability and wear resistance. Hence, initial morphological adaptation and post-brushing volumetric loss should be evaluated together.

The present in vitro study aimed to assess the morphological adaptation of restorations performed using the stamp technique with different composite resins and to quantitatively evaluate brushing-induced volumetric loss and surface morphological alterations using OraCheck software. The first null hypothesis was that composite resins with different viscosities used with the stamp technique would not differ in terms of morphological adaptation. The second null hypothesis was that composite resins with different viscosities used with the stamp technique would not differ in terms of wear after toothbrushing simulation.

## 2. Materials and Methods

### 2.1. Sample Selection and Baseline Digital Acquisition

Sample size was determined a priori using G*Power (version 3.1.9.7; Heinrich Heine University Düsseldorf, Düsseldorf, Germany) for a one-way ANOVA design with four independent groups (α = 0.05, two-tailed). Based on comparable in vitro studies evaluating volumetric differences among composite resin groups under toothbrushing simulation, a large effect size was anticipated. Using Cohen’s *f* = 0.50, consistent with established large-effect conventions and with the magnitude of volumetric differences reported in analogous dental wear studies, the analysis indicated a minimum required sample size of 14 specimens per group (total *N* = 56) to achieve approximately 85% power [27]. To provide an additional margin of safety, 15 specimens were allocated to each of the four experimental groups, yielding a total of 60 standardized extracted molars and an estimated achieved power of 89.6% (λ = 15.00, critical *F*(3, 56) = 2.769).

This in vitro study was conducted using sixty standardized mandibular first molar model teeth (ERAY Dental, Istanbul, Turkey). Before any experimental procedures, each specimen was individually scanned with an intraoral scanner (CEREC Omnicam, Dentsply Sirona, Bensheim, Germany). The digital data obtained during intraoral scanning were directly imported into the three-dimensional evaluation software (OraCheck, Cyfex AG, Zurich, Switzerland) for subsequent analysis. These datasets were used as baseline reference models, representing the original occlusal surface morphology of each specimen before the restorative procedures, and were defined as T0. The scans obtained after restoration and after toothbrushing simulation were defined as T1 and T2, respectively.

All scanning procedures were performed by a single operator under standardized lighting and scanning conditions to minimize operator-dependent variability. During digital acquisition, each model was embedded in silicone putty (Oxasil, Kulzer, Hanau, Germany) to maintain a stable and standardized position, thereby minimizing movement during the scanning procedure.

**Figure 1:**
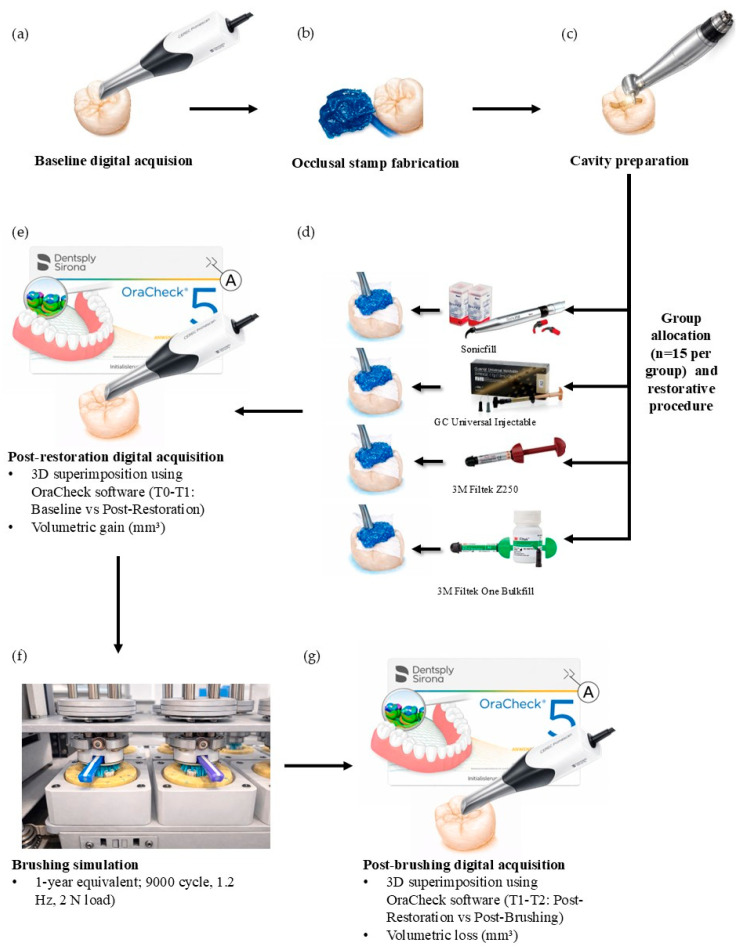
Schematic illustration of the experimental workflow: (**a**) Baseline digital acquisition; (**b**) Occlusal stamp fabrication; (**c**) Cavity preparation; (**d**) Group allocation and restorative procedures; (**e**) Post-restoration digital acquisition and superimposition analysis; (**f**) Toothbrushing simulation; (**g**) Post-toothbrushing digital acquisition and wear analysis.

The overall experimental workflow is illustrated in Figure 1, including baseline digital acquisition (Figure 1a), occlusal stamp fabrication (Figure 1b), cavity preparation (Figure 1c), group allocation and restorative procedures (Figure 1d), post-restoration digital acquisition (Figure 1e), brushing simulation (Figure 1f), and post-brushing digital acquisition (Figure 1g).

### 2.2. Specimen Preparation and Restoration Procedure

Before cavity preparation, the occlusal anatomy of each specimen was recorded using a light-cured gingival barrier material (OpalDam, Ultradent Products Inc., South Jordan, UT, USA) to obtain occlusal stamp impressions. This material was selected because of its ability to accurately reproduce occlusal morphology and its controlled polymerization characteristics, which facilitate removal while maintaining dimensional stability [28]. The material was applied to the dried occlusal surface in an amount sufficient to completely cover the occlusal anatomy of each specimen, thereby standardizing the material quantity. A micro-brush handle was embedded as a holder, and light curing was performed using an LED curing unit (D-Light Pro, GC Corporation, Tokyo, Japan).

Following occlusal stamp fabrication, standardized Class I cavities with a depth of 2 mm were prepared on the occlusal surfaces of all specimens. The cavity dimensions were standardized using cavity preparation burs under controlled conditions, and the cavity depth was verified with a calibrated periodontal probe to ensure consistency among all samples. The cavity preparation burs were replaced with new ones after every five cavity preparations. After cavity preparation, the specimens were randomly divided into four experimental groups (*n* = 15) according to the restorative material used. The evaluated restorative materials, their compositions, and manufacturers are presented in Table 1.

- Group 1: Sonic-activated bulk-fill composite resin (SonicFill 3, Kerr Corporation, Orange, CA, USA).
- Group 2: Highly filled injectable composite resin (G-ænial Universal Injectable, GC Corporation, Tokyo, Japan).
- Group 3: Conventional hybrid composite resin (Filtek Z250, 3M ESPE, St. Paul, MN, USA).
- Group 4: Conventional bulk-fill composite resin (Filtek One Bulk Fill, 3M ESPE, St. Paul, MN, USA).

All restorative procedures were performed according to the manufacturers’ instructions.

The assigned composite resin was placed into the cavity according to the protocol of each experimental group. Since the present study was conducted on artificial teeth and aimed to evaluate the morphological reproduction of restorations rather than adhesive bonding performance, no adhesive system was applied, similar to a previous in vitro study performed on typodont models [29]. A thin Teflon tape was then positioned over the uncured composite to prevent adhesion to the stamp, and the previously prepared occlusal stamp was applied to reproduce the original occlusal anatomy. Gentle pressure was applied to allow excess material to flow out, and any excess composite was carefully removed before polymerization.

In the SonicFill group, the composite resin was delivered into the cavity using the manufacturer’s dedicated sonic activation handpiece, which temporarily lowers the viscosity of the material during placement to improve adaptation to the cavity walls. Following placement, the occlusal anatomy was shaped using the occlusal stamp in the same manner as described above. Polymerization was performed at an output intensity of 1200 mW/cm^2^ for 20 s using an LED curing unit connected to a power supply to ensure stable light output. All restorative procedures were performed by a single experienced operator. To preserve the reproduced occlusal morphology, no occlusal adjustment, finishing, or polishing procedures were carried out in any of the study groups [30], as these procedures may alter the reproduced anatomy and introduce additional operator-dependent variables.

### 2.3. Three-Dimensional Assessment After Restoration

After polymerization, each specimen was rescanned 24 h later using the same intraoral scanner (CEREC Omnicam, Dentsply Sirona, Bensheim, Germany) under identical scanning conditions as the baseline acquisition. The 24 h interval was selected to allow complete polymerization of the composite restorations produced using the stamp technique. These datasets were defined as T1.

The postoperative datasets were imported into OraCheck software and superimposed onto the baseline datasets using a best-fit alignment protocol. The alignment process was accepted when the registration reliability was ≥95% and the average surface deviation was approximately 0.03 µm. This three-dimensional comparison enabled quantitative volumetric analysis of the restorations created using the stamp technique.

Additionally, cross-sectional views generated by the software were used for qualitative observation of morphological adaptation and anatomical conformity with the original preoperative occlusal anatomy. During all scanning procedures, each specimen was maintained in a stable positioning setup to ensure consistent orientation.

### 2.4. Toothbrushing Simulation

Following baseline digital evaluation, all restored specimens were subjected to a standardized toothbrushing simulation to assess brushing-related wear resistance. A toothbrushing simulator was used to represent approximately one year of clinical toothbrushing under a constant load of 2.0 N, with a brushing frequency of 1.2 Hz and a total of 9000 brushing cycles. The applied load of 2.0 N was selected based on previously reported toothbrushing simulation protocols [31]. The cycle count was determined based on a previously reported model in which 5000 cycles performed at 1 Hz were considered equivalent to approximately six months of clinical toothbrushing [32]. Considering the higher brushing frequency used in the present study (1.2 Hz), the selected brushing regimen was considered to represent approximately one year of clinical toothbrushing. Brushing was performed using a linear reciprocating motion with a 10 mm stroke length and medium-bristle toothbrush heads (Oral-B Pro-Expert Medium, Procter & Gamble, Schwalbach am Taunus, Germany).

A toothpaste slurry was prepared using a dentifrice with a Relative Dentin Abrasivity (RDA) value of 70 (Colgate Total Original, Colgate-Palmolive Company, New York, NY, USA) at a ratio of 2 g distilled water to 1 g toothpaste, in accordance with ISO/TR 14569-1:2007 [33]. Throughout the simulation, the specimens were kept in distilled water to prevent dehydration, and residual slurry was thoroughly rinsed after the brushing procedure.

### 2.5. Three-Dimensional Assessment After Toothbrush Simulation

After the brushing simulation, all specimens were rinsed and dried using an air–water spray. Upon completion of this procedure, all specimens were rescanned using the same scanning protocol. These datasets were defined as T2.

The datasets obtained at three time points (T0, T1, and T2) were imported into OraCheck software and analyzed using surface-matching algorithms. Two comparative analyses were performed. The first comparison between T0 and T1 datasets was used to evaluate the volumetric difference between the original occlusal morphology and the restored surface created using the stamp technique. The second comparison between T1 and T2 datasets was conducted to quantify brushing-related volumetric material loss.

Volumetric changes were calculated using the volumetric analysis function of the software and expressed in cubic millimeters (mm^3^). In addition, the average distance value (µm) provided by the software was recorded as an indicator of the agreement and reliability of the dataset superimposition.

### 2.6. Statistical Analysis

All statistical analyses were performed using SPSS Statistics (version 26.0; IBM Corp., Armonk, NY, USA). Complementary computations and visualizations were conducted in Python (version 3.11; Python Software Foundation, Wilmington, DE, USA) using the SciPy (version 1.13.0), NumPy (version 1.26.4), and Matplotlib (version 3.8.4) libraries. The significance level was set at α = 0.05 for all tests. Prior to inferential analysis, the normality of each group’s distribution was assessed using the Shapiro–Wilk test (*n* = 15 per group), and homogeneity of variance across groups was evaluated with Levene’s test. Normality and variance assumptions jointly informed the choice between parametric and non-parametric inferential procedures.

For the T0–T1 volumetric gain outcome, all groups satisfied normality (Shapiro–Wilk, all *p* > 0.05) and variance homogeneity (Levene’s test: F = 1.848, *p* = 0.149). Accordingly, a one-way ANOVA was applied, followed by Tukey’s Honestly Significant Difference (HSD) post hoc test for pairwise comparisons. Effect size was quantified as eta-squared (η^2^); Cohen’s d was reported for each pairwise contrast.

For the T1–T2 volumetric loss outcome, Levene’s test indicated significant heterogeneity of variance (F = 3.196, *p* = 0.030), precluding the use of parametric ANOVA. The Kruskal–Wallis H test was therefore employed, with Dunn’s post hoc test and Bonferroni correction applied to all six pairwise comparisons (adjusted threshold: *p* < 0.0083). Effect size was expressed as epsilon-squared (ε^2^); rank-biserial correlation (r) was reported for each pairwise contrast.

The relationship between T0–T1 volumetric gain (reflecting occlusal morphological fidelity following stamp restoration) and T1–T2 volumetric loss (reflecting wear susceptibility following brushing simulation) was examined using Spearman’s rank correlation coefficient (*r_s_*). Given that lower T0–T1 values indicate closer replication of the original occlusal morphology, correlations were computed separately for each group (*n* = 15) to assess whether specimens with higher morphological fidelity also exhibited differential wear behavior within each composite type.

## 3. Results

### 3.1. Descriptive Statistics and Assumption Testing

Shapiro–Wilk tests confirmed that all groups met normality for both outcome variables (T0–T1: all W ≥ 0.882, all *p* ≥ 0.050; T1–T2: all W ≥ 0.888, all *p* ≥ 0.062). Levene’s test indicated homogeneous variances for T0–T1 (F = 1.848, *p* = 0.149), but significant heterogeneity for T1–T2 (F = 3.196, *p* = 0.030), informing the selection of different inferential approaches for the two outcomes.

Descriptive statistics for T0–T1 volumetric gain and T1–T2 volumetric loss are presented in Table 2.

### 3.2. T0–T1 Volumetric Gain: Comparisons of Groups

**Figure 2:**
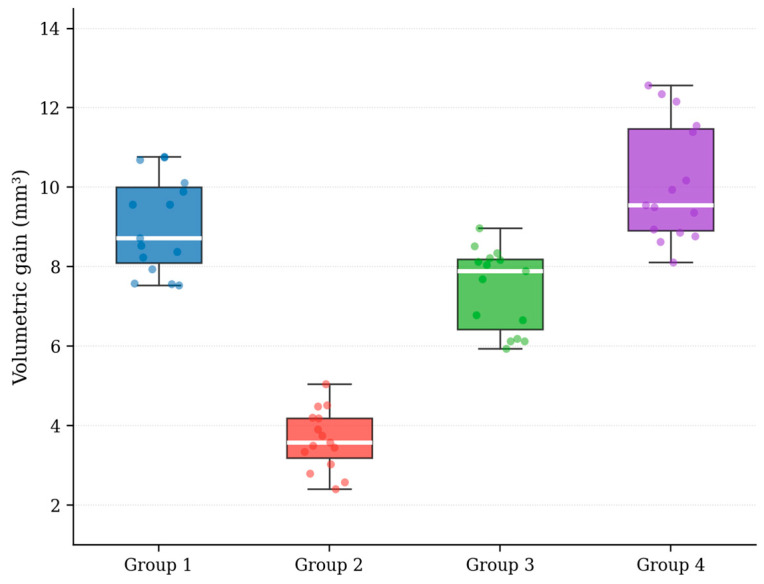
Box plots depicting T0–T1 volumetric gain (mm^3^) by experimental group.

**Figure 3:**
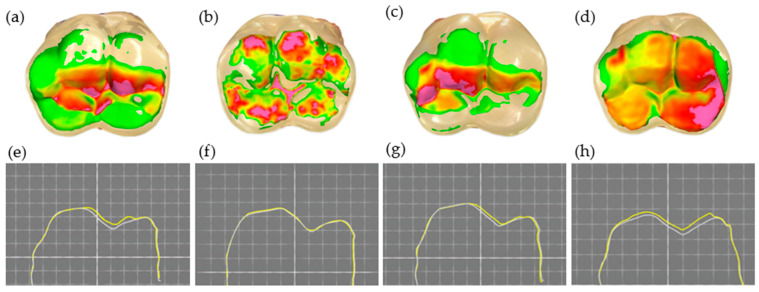
Representative 3D deviation color maps (**a**–**d**) and corresponding cross-sectional profile curves (**e**–**h**) obtained from one specimen per group at T1 following T0–T1 superimposition. Green indicates minimal deviation, whereas warm and purple colors indicate positive and negative deviations, respectively.

One-way ANOVA revealed a statistically significant overall difference in T0–T1 volumetric gain among the four groups (*F*(3, 56) = 90.63, *p* < 0.001, η^2^ = 0.829), indicating a large effect. Post hoc comparisons using Tukey’s HSD demonstrated significant pairwise differences between all group combinations (all *p* ≤ 0.004). Group 2 exhibited the lowest volumetric gain, indicating the greatest morphological fidelity to the pre-restoration occlusal surface, and was significantly different from all other groups. Group 4 demonstrated the highest volumetric gain, reflecting the greatest departure from the original morphology, and likewise differed significantly from all remaining groups (Table 3; Figure 2). Representative color maps and corresponding cross-sectional profiles for each experimental group are shown in Figure 3a–d and Figure 3e–h, respectively.

### 3.3. T1–T2 Volumetric Loss: Comparisons of Groups

**Figure 4:**
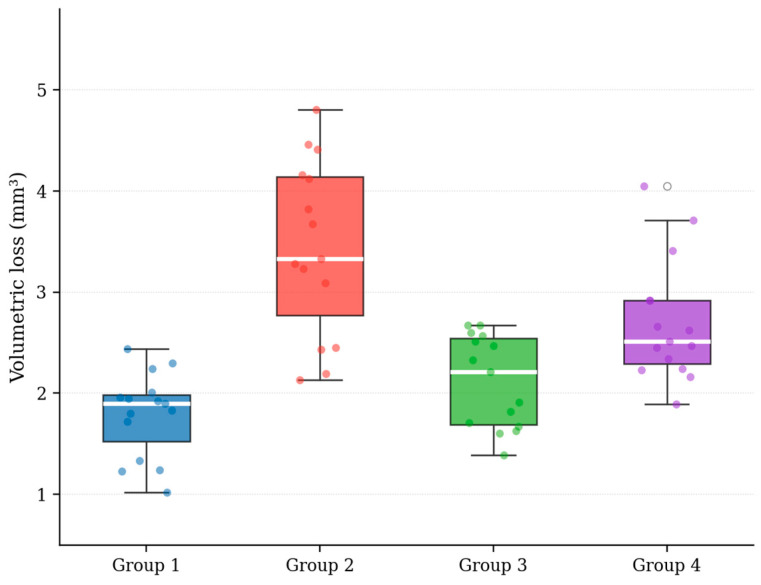
Box plots depicting T1–T2 volumetric loss (mm^3^) by experimental group.

The Kruskal–Wallis test demonstrated a statistically significant difference in T1–T2 volumetric loss among the four groups (*H*(3) = 29.54, *p* < 0.001, ε^2^ = 0.474), indicating a large effect. Dunn’s post hoc test with Bonferroni correction identified significant differences between Group 1 and Group 2 (*p* < 0.001, *r* = 0.938), Group 1 and Group 4 (*p* = 0.003, *r* = 0.844), and Group 2 and Group 3 (*p* = 0.002, *r* = 0.751). No statistically significant differences were observed between Groups 1 and 3, Groups 2 and 4, or Groups 3 and 4 following Bonferroni correction (all *p* > 0.05). Group 2 exhibited the greatest volumetric loss, while Group 1 showed the least (Table 4; Figure 4).

### 3.4. Relationship Between Occlusal Adaptation and Wear Susceptibility

**Figure 5:**
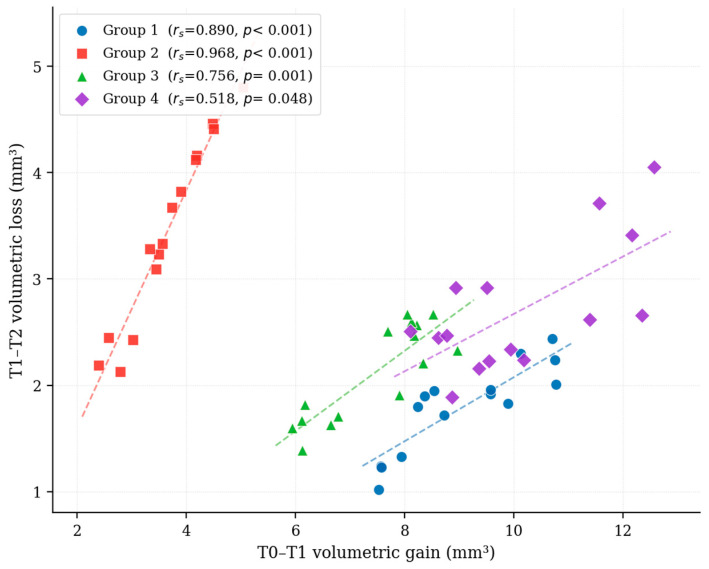
Relationship between T0–T1 volumetric gain and T1–T2 volumetric loss across experimental groups.

Within-group correlations revealed strong and statistically significant positive associations between T0–T1 volumetric gain and T1–T2 volumetric loss in all four groups (Table 5; Figure 5). Specimens within a given group that exhibited lower volumetric gain, and thus higher morphological fidelity following stamp restoration, also tended to show lower volumetric loss after brushing simulation, and vice versa. This within-group pattern was most pronounced in Group 2 (*r_s_* = 0.968, *p* < 0.001) and Group 1 (*r_s_* = 0.890, *p* < 0.001) and remained significant in Groups 3 and 4. It is noteworthy that the absence of a significant pooled correlation across all 60 specimens reflects the dominant between-group variance driven by differences in composite material type, which obscures the within-group relationship when data are aggregated.

## 4. Discussion

The biomimetic approach aims to replicate the structure and function of natural systems while preserving sound tooth structure as much as possible, in accordance with the principle of minimal intervention [1,34]. Composite resin materials enable the reconstruction of natural tooth morphology and the establishment of functional occlusion [35]. Achieving accurate occlusal topography is critical for functional success for direct composite restorations. The stamp technique, proposed for this purpose, represents a biomimetic approach by allowing the direct transfer of preoperative occlusal morphology to the restorative material [36]. This technique is indicated in carefully selected clinical scenarios, particularly posterior carious or erosive lesions in which the occlusal surface and cusp morphology remain largely intact [37]. In this technique, morphological accuracy depends not only on the application procedure but also on the viscoelastic behavior of the material [38]. Due to their pseudoplastic nature, composite resins exhibit reduced viscosity under applied pressure, which facilitates improved adaptation to the surrounding surfaces [39].

The flowability and viscosity of composite resins are key parameters influencing the success of the stamp technique. While highly viscous materials may lead to surface deformation, low-viscosity materials may exhibit collapse after removal of the stamp [30]. Therefore, the rheological properties of the material are critical not only for achieving morphological accuracy but also for maintaining this morphology over time. Accordingly, the present study evaluated the influence of composite resins with different viscosities on the morphological accuracy of restorations fabricated using the stamp technique.

In response to increasing clinical demands for easier handling, improved adaptation, and enhanced restorative performance, manufacturers have focused not only on optimizing the formulations of flowable composites, but also on developing alternative viscosity-modification approaches such as sonic-activated and thermoviscous composite systems [15,40]. These developments include modifications in filler content, particle size, and monomer systems, which enhance both the adaptation ability and the mechanical and physical properties of the materials [15]. Furthermore, preheating has been reported to reduce composite viscosity and improve material flow and adaptation to cavity walls [41]. In the present study, the significantly lower volumetric change observed in the GC Injectable composite (Group 2) compared to the other groups indicates that this material was able to reproduce occlusal morphology with the highest accuracy. This finding may be attributed to the high flowability of injectable composites, which allows more effective adaptation to both cavity walls and the stamp impression, enabling a more precise transfer of surface topography. Dionysopoulos et al. reported that methods increasing composite flowability, such as preheating and sonic activation, improve internal adaptation and reduce void formation [42]. Similarly, Baltacıoğlu et al. [43] and Scepanovic et al. [44] demonstrated that low-viscosity composites exhibit superior adaptation performance.

In the present study, the Filtek Z250 composite (Group 3) demonstrated higher morphological accuracy than the SonicFill (Group 1) and Filtek One Bulk Fill (Group 4) composites. This finding may be associated with the balanced rheological properties of conventional hybrid composites, which exhibit intermediate viscoelastic profiles positioned between flowable and condensable formulations [38,45]. Assiri et al. reported that although no statistically significant difference in marginal accuracy was observed between bulk-fill composites and Filtek Z250, the Z250 group showed numerically higher adaptation values and a more favorable trend [46]. Similarly, Soares et al. demonstrated that the marginal integrity of Filtek Z250 was comparable to that of the latest-generation bulk-fill materials and remained stable even after aging [47]. However, contrary to the findings of the present study, Kantovitz et al. reported that high-viscosity composites exhibited better adaptation than low-viscosity materials [48]. This difference may be related to differences in cavity depth and the more pronounced polymerization shrinkage stresses observed in natural tooth structures. In the present study, stamp technique restorations were performed in standardized 2 mm cavities using phantom teeth, which may have limited shrinkage-related stresses and favored more accurate surface reproduction by low-viscosity materials.

When the morphological adaptation performance of the materials was evaluated, SonicFill demonstrated lower volumetric gain compared to Filtek One Bulk Fill following stamp restoration, indicating more accurate reproduction of occlusal morphology. However, both bulk-fill composites showed inferior morphological accuracy compared to GC Injectable and Filtek Z250. Sonic activation increases composite temperature during application, reducing viscosity and improving adaptation to cavity walls [49]. However, the rapid recovery of viscosity after cessation of sonic activation should be considered [44]. Andrade et al. reported that sonic activation reduces adaptation defects in SonicFill, whereas Filtek One Bulk Fill may exhibit more pronounced errors due to the absence of viscosity modulation. Specifically, sonic energy transferred to SonicFill during application resulted in an 87% reduction in viscosity [50]. This improved flowability has been attributed to the presence of a rheological modifier and diluted TEGDMA monomer in its composition, which facilitate temporary viscosity reduction under sonic activation [51]. However, once sonication ceased, the material immediately returned to its baseline rheological state, suggesting that the rheological advantage is transient and limited to the moment of application [50]. Despite the use of sonic activation, the thixotropic recovery of SonicFill and the inherently high viscosity of Filtek One Bulk Fill may have limited their ability to fully reproduce occlusal details under stamp pressure.

In addition to the viscosity-modification strategies employed by SonicFill, the superior morphological adaptation observed for G-ænial Universal Injectable may also be explained by its rheological characteristics. According to manufacturer documentation, G-ænial Universal Injectable combines a low-viscosity injectable formulation with enhanced thixotropic behavior, which promotes material adaptation while maintaining shape stability during placement [52]. Furthermore, rheological investigations have demonstrated higher complex viscosity values for bulk-fill composites than for conventional hybrid composites under comparable testing conditions [53]. Together, these findings suggest that viscosity-related material behavior may contribute to differences in morphological reproduction observed in the stamp technique. However, because the rheological properties of the tested materials were not directly measured, these interpretations should be considered potential mechanisms rather than definitive causal relationships.

Following toothbrushing simulation, the results revealed differences in the mechanical durability and wear resistance of the materials. The highest volumetric loss was observed in the G-ænial Universal Injectable group, whereas the lowest was observed in the SonicFill group. Filtek One Bulk Fill and Filtek Z250 exhibited intermediate values, with no statistically significant difference between them. In the study by Yang et al., which evaluated the wear resistance of different composite materials, SonicFill was found to exhibit the most stable performance. The authors attributed this result to the high filler content of the material, which protects the resin matrix and prevents particle dislodgement from the surface [54]. Similarly, in a study conducted by Alkhudhairy, SonicFill was reported to exhibit the lowest surface loss among various bulk-fill composites, particularly when polymerized under low light intensity [55].

Although some studies have reported no significant differences in surface roughness between SonicFill 2 and Filtek Bulk Fill Posterior composites after long-term brushing simulations [56], the present study demonstrated significantly higher wear resistance for SonicFill 2 compared to Filtek Bulk Fill Posterior. This difference may be attributed to differences in brushing protocols or to the improved filler–matrix interaction in the newer SonicFill formulation. Furthermore, previous studies have suggested that bulk-fill composites generally exhibit lower wear resistance than conventional composites, although their behavior may vary depending on filler characteristics and distribution [57].

However, contrary to the findings of the present study, Shimokawa et al. reported no statistically significant difference in surface roughness between SonicFill 2 and Filtek Bulk Fill Posterior after 25,000 brushing cycles [56]. In contrast, in our study, SonicFill exhibited significantly higher wear resistance than the Filtek One Bulk Fill group. This difference may be attributed to the long-term brushing simulation applied by Shimokawa et al., involving 25,000 cycles (approximately 2 years), which may have affected the fatigue resistance of the materials differently, or to the structural advantages provided by the optimized filler–matrix interaction in the newer SonicFill 3 formulation used in the present study. Although Osiewicz et al. reported that bulk-fill composites generally exhibit lower wear resistance than conventional composites, they also emphasized that this material group is not homogeneous and that differences in filler content and distribution significantly influence wear behavior [57]. In this context, despite being a bulk-fill material, the higher filler content of SonicFill may explain its lower volumetric loss compared to Filtek One Bulk Fill. In our study, although the conventional composite Filtek Z250 showed numerically lower wear than Filtek One Bulk Fill, this difference was not statistically significant. Similarly, a study evaluating composites subjected to attrition wear in a chewing simulator reported that Filtek Z250 exhibited wear resistance comparable to bulk-fill composites, with no significant differences among groups [58]. Nevertheless, the numerically lower volumetric loss of Z250 compared to Filtek One Bulk Fill, although not statistically significant, suggests that wear behavior is not solely dependent on the bulk-fill or conventional classification but is also influenced by material properties such as filler content, particle size, and filler–matrix interaction. In this regard, the higher filler content of Filtek Z250 may have contributed to improved protection of the resin matrix and enhanced wear resistance compared to Filtek One Bulk Fill.

Recent studies have reported notable improvements in the mechanical properties and wear resistance of flowable composites [59]. When the wear behavior of these materials is examined, particular attention has been given to studies comparing highly filled flowable composites, such as G-ænial Universal Injectable, with conventional composites. Ozdemir et al., high-filled flowable composites evaluated using a chewing simulator exhibited greater wear and lower surface hardness compared to conventional composites, such as Filtek Z250 [60]. These findings suggest that flowable composites may have lower wear resistance compared to conventional materials, which is consistent with the higher volumetric loss observed in the G-ænial Universal Injectable group in the present study. This may be associated with the relatively lower surface hardness of flowable composites and the influence of filler–matrix interactions on wear behavior. Although flowable composites have been improved by increasing their filler content, the present findings suggest that they may still exhibit a tendency toward lower wear resistance compared to conventional composites. It has also been reported that the wear performance of highly filled flowable composites may vary depending on polymerization time and that, when adequately polymerized, they can demonstrate wear resistance comparable to conventional composites [14]. However, the higher volumetric loss observed in the G-ænial Universal Injectable group in the present study suggests that the wear behavior of flowable composites was influenced not only by filler content but also by material composition and application conditions.

One of the most notable findings of this study is that the materials exhibited different performances in terms of morphological accuracy and wear resistance. GC Injectable demonstrated the highest morphological accuracy following the stamp technique, but also the greatest wear after the toothbrushing simulation. In contrast, SonicFill showed low morphological accuracy but superior wear resistance. When all groups were evaluated together, no significant relationship was found between morphological adaptation and wear; however, a consistent trend was observed within each group. Specifically, within the same material, specimens exhibiting higher volumetric gain (i.e., lower morphological accuracy) tended to show greater wear. These findings indicate that morphological accuracy may influence not only esthetic and functional outcomes but also wear behavior. In this context, achieving more accurate occlusal morphology through the stamp technique may contribute to the improved surface stability of restorations. Furthermore, these results suggest that the relationship between morphological accuracy and mechanical durability is material-dependent and may not be generalizable across different composite systems.

Although finishing and polishing procedures were intentionally omitted in the present study to preserve the reproduced occlusal morphology and avoid introducing operator-dependent variables, it should be acknowledged that these procedures are routinely performed in clinical practice. Restorations exhibiting greater morphological discrepancies following stamp reproduction would be expected to require more extensive occlusal adjustment, finishing, and polishing. Therefore, the degree of morphological discrepancy observed in the present study may serve as an indirect indicator of the additional chairside time and clinical effort potentially required for each restorative material.

From a clinical perspective, an ideal restorative material should provide mechanical strength and maintain these properties over time, as clinical performance is closely associated with wear resistance and long-term durability [61,62]. However, none of the materials evaluated in the present study were able to achieve both optimal morphological accuracy and wear resistance simultaneously. This finding indicates that further improvements are necessary to achieve both accurate reproduction and long-term preservation of occlusal morphology.

In addition, the three-dimensional analysis method used in this study enabled objective and quantitative evaluation of morphological accuracy and volumetric changes. Previous studies have demonstrated that three-dimensional superimposition techniques provide a reliable and effective approach for assessing tooth wear and are applicable in clinical settings [63]. Various software programs, such as MeshLab, CloudCompare, and Geomagic ControlX, have been used for this purpose [64,65,66]. However, OraCheck was preferred in the present study due to its compatibility with the CEREC. Such digital analysis methods are expected to become increasingly widespread in the evaluation of restorative material performance.

This study has several limitations that should be considered when interpreting the findings. Firstly, the use of phantom teeth does not fully reflect the complex anatomy of natural teeth; similarly, phantom teeth cannot fully replicate the structural and biological properties of enamel and dentine. Therefore, the application of an adhesive system was not required, as no bonding to dental tissues was intended. As a result, it is not possible for these findings to fully replicate clinical practice. In addition, as an in vitro study, the biological and physicochemical conditions of the oral environment, including thermal conditions, masticatory loading, salivary activity, toothbrushing abrasion, and enzymatic degradation, were not fully reproduced. These factors may influence the surface integrity and wear behavior of restorative materials over time, thereby limiting the direct clinical extrapolation of the findings. Another limitation of the present study is that surface roughness and hardness, which may influence the wear behavior of resin composites, were not evaluated. Therefore, their contribution to the observed wear patterns could not be determined. Future studies combining volumetric wear analysis with surface roughness and hardness measurements may provide a more comprehensive understanding of wear mechanisms. Wear analysis was limited to toothbrushing simulation, and other wear mechanisms such as attrition and erosion were not evaluated. In addition, finishing and polishing procedures were intentionally omitted to preserve the reproduced occlusal morphology and to avoid the introduction of operator-dependent variables. However, because these procedures are routinely performed in clinical practice and may influence restoration morphology and wear behavior, the findings should be interpreted with caution when extrapolating them to clinical situations. Furthermore, patient-based clinical parameters were beyond the scope of this study. One limitation of this study is that the rheological properties of the tested materials were not directly evaluated. Future studies incorporating direct rheological analysis are needed to better clarify the influence of material viscosity on morphological adaptation and wear behavior. Further studies should be conducted on natural teeth using long-term, multifactorial simulation models that incorporate different wear mechanisms and include clinical outcomes.

## 5. Conclusions

Within the limitations of this in vitro study, composite materials with different rheological properties showed differences depending on the material in morphological accuracy and wear resistance when applied using the stamp technique. The highly filled injectable composite resin (G-ænial Universal Injectable) achieved the highest morphological accuracy but exhibited the greatest wear. The lowest morphological accuracy was observed in Filtek One Bulk Fill. SonicFill demonstrated lower morphological accuracy but superior wear resistance.

Although no significant correlation was observed across all groups, restorations with higher morphological accuracy consistently tended to exhibit lower wear within each material group. These findings suggest that morphological accuracy may influence not only esthetic and functional outcomes but also the long-term surface stability of restorations. From a biomimetic perspective, achieving a balance between accurate morphology and mechanical durability remains a challenge for current composite resin systems used in biomimetic restorative approaches.

## Figures and Tables

**Table 1:** Composition and manufacturer information of the materials used in this study.

Restorative Material	Type	Composition	Lot No	Manufacturer
SonicFill 3	Sonic-activated bulk-fill composite	Filler content (wt%): 83.5 Matrix: Bis-GMA, TEGDMA, Bis-EMA Inorganic filler: SiO_2_ glass, oxide	9591358	Kerr Corporation, Orange, CA, USA
Filtek One Bulk Fill	Conventional bulk-fill composite	Filler content (wt%): 76.5 Matrix: AUDMA, AFM, DDMA, UDMA Inorganic filler: Ytterbium trifluoride, zirconia/silica	10639667	3M ESPE, St. Paul, MN, USA
Filtek Z250	Universal composite resin	Filler content (wt%): 82.5 Matrix: Bis-GMA, UDMA, Bis-EMA Inorganic filler: Zirconia/silica	11500542	3M ESPE, St. Paul, MN, USA
G-ænial Universal Injectable	Highly filled injectable composite	Filler content (wt%): 69 Matrix: UDMA-based matrix Inorganic filler: Barium–aluminum–borosilicate glass, silica	250528A	GC Corporation, Tokyo, Japan
OpalDam	Gingival barrier	UDMA, TEGDMA, barium glass, SiO_2_, CQ, DMAEMA, pigments, photoinitiators	1222913	Ultradent Products, South Jordan, UT, USA

**Table 2:** Descriptive statistics for T0–T1 volumetric gain and T1–T2 volumetric loss across experimental groups (*n* = 15 per group).

	T0–T1: Volumetric Gain (mm^3^)				T1–T2: Volumetric Loss (mm^3^)			
	Group 1	Group 2	Group 3	Group 4	Group 1	Group 2	Group 3	Group 4
n	15	15	15	15	15	15	15	15
Mean ± SD	9.057 ± 1.205	3.650 ± 0.758	7.453 ± 1.033	10.125 ± 1.493	1.793 ± 0.418	3.438 ± 0.866	2.117 ± 0.456	2.705 ± 0.604
Median	8.720	3.570	7.900	9.550	1.900	3.330	2.210	2.510
Min–Max	7.53–10.77	2.40–5.05	5.94–8.97	8.11–12.57	1.02–2.44	2.13–4.80	1.39–2.67	1.89–4.05
Q1–Q3	8.090–10.005	3.185–4.190	6.415–8.195	8.905–11.475	1.525–1.985	2.770–4.140	1.690–2.540	2.290–2.920

SD: standard deviation; Q1–Q3: first–third quartile. T0–T1 values represent net volumetric gain (mm^3^) following stamp restoration relative to baseline; lower values indicate closer replication of the original occlusal morphology. T1–T2 values represent absolute volumetric loss (mm^3^) following toothbrushing simulation.

**Table 3:** Pairwise post hoc comparisons for T0–T1 volumetric gain (one-way ANOVA with Tukey’s HSD).

Comparison	Mean Diff. (mm^3^)	q	*p* (Tukey)	Cohen’s d	Significance
Group 1 vs. Group 2	+5.407	18.154	<0.001	5.37	***
Group 1 vs. Group 3	+1.605	5.387	<0.001	1.43	***
Group 1 vs. Group 4	−1.068	3.586	0.004	0.79	**
Group 2 vs. Group 3	−3.803	12.767	<0.001	4.20	***
Group 2 vs. Group 4	−6.475	21.740	<0.001	5.47	***
Group 3 vs. Group 4	−2.673	8.973	<0.001	2.08	***

F(3, 56) = 90.63, *p* < 0.001, η^2^ = 0.829 (large effect). Mean difference: positive values indicate the first group exceeds the second. Cohen’s d: 0.2 = small, 0.5 = medium, 0.8 = large. *** *p* < 0.001; ** *p* < 0.01.

**Table 4:** Pairwise post hoc comparisons for T1–T2 volumetric loss (Kruskal–Wallis with Dunn’s test, Bonferroni correction).

Comparison	z	*p* (Bonf.)	r (Rank-Biserial)	Significance
Group 1 vs. Group 2	5.008	<0.001	0.938	***
Group 1 vs. Group 3	1.417	0.940	0.333	ns
Group 1 vs. Group 4	3.528	0.003	0.844	**
Group 2 vs. Group 3	3.591	0.002	0.751	**
Group 2 vs. Group 4	1.479	0.834	0.453	ns
Group 3 vs. Group 4	2.112	0.208	0.493	ns

H(3) = 29.54, *p* < 0.001, ε^2^ = 0.474 (large effect). *p* values are Bonferroni-corrected for six pairwise comparisons (adjusted threshold: *p* < 0.0083). r: rank-biserial correlation coefficient. *** *p* < 0.001; ** *p* < 0.01; ns: not significant.

**Table 5:** Within-group Spearman rank correlations between T0–T1 volumetric gain and T1–T2 volumetric loss (*n* = 15 per group).

	r_s_	*p*	n	Direction
Group 1	0.890	<0.001	15	Positive ***
Group 2	0.968	<0.001	15	Positive ***
Group 3	0.756	0.001	15	Positive ***
Group 4	0.518	0.048	15	Positive *

r_s_: Spearman rank correlation coefficient. A positive direction indicates that higher volumetric gain is associated with greater volumetric loss within the same group. *** *p* < 0.001; * *p* < 0.05.

## Data Availability

The raw data supporting the conclusions of this article will be made available by the authors on request.

## References

[1] Singer L., Fouda A., Bourauel C. (2023). Biomimetic approaches and materials in restorative and regenerative dentistry. BMC Oral Health.

[2] Mary G., Jayadevan A. (2016). Microbrush stamp technique to achieve occlusal topography for composite resin restorations-A Technical Report. J. Sci. Dent..

[3] Müller H.C. (2010). Registration of occlusion by buccal scan in Cerec software version 3.80. Int. J. Comput. Dent..

[4] Pawar L.P., Patel A., Chandak M., Wazurkar S., Nadgouda M. (2024). Perfecting the Craft: Composite Restoration Elevated with the Stamp Technique. Cureus.

[5] Kun Q., Wang Q.L., Jie P. (2021). 3D Digital Evaluation for Direct Composite Restoration Using the Modified Stamp Technique. Chin. J. Dent. Res..

[6] Chatzidimitriou K., Katechi V., Seremidi K. (2025). Innovative Composite Resin Restoration Techniques in Posterior Permanent Teeth of Young Patients: Presentation of Two Clinical Cases. Case Rep. Dent..

[7] Alshehadat S.A., Halim M.S., Carmen K., Fung C.S. (2016). The stamp technique for direct Class II composite restorations: A case series. J. Conserv. Dent. Endod..

[8] Saoji S.S., Ikhar A., Manik K., Awghad S., Panchal S. (2024). Elevating Restorative Dentistry: Use of the Art of Stamp Techniques in Mandibular Posterior Regions. Cureus.

[9] Zafar M.S., Amin F., Fareed M.A., Ghabbani H., Riaz S., Khurshid Z., Kumar N. (2020). Biomimetic aspects of restorative dentistry biomaterials. Biomimetics.

[10] Bellinaso M.D., Soares F.Z.M., Rocha R.O. (2019). Do bulk-fill resins decrease the restorative time in posterior teeth? A systematic review and meta-analysis of in vitro studies. J. Investig. Clin. Dent..

[11] Ilie N. (2022). Resin-Based Bulk-Fill Composites: Tried and Tested, New Trends, and Evaluation Compared to Human Dentin. Materials.

[12] Leprince J.G., Palin W.M., Vanacker J., Sabbagh J., Devaux J., Leloup G. (2014). Physico-mechanical characteristics of commercially available bulk-fill composites. J. Dent..

[13] Vouvoudi E.C. (2022). Overviews on the Progress of Flowable Dental Polymeric Composites: Their Composition, Polymerization Process, Flowability and Radiopacity Aspects. Polymers.

[14] Checchi V., Generali L., Corciolani L., Breschi L., Mazzitelli C., Maravic T. (2025). Wear and roughness analysis of two highly filled flowable composites. Odontology.

[15] Tzimas K., Pappa E., Fostiropoulou M., Papazoglou E., Rahiotis C. (2025). Highly Filled Flowable Composite Resins as Sole Restorative Materials: A Systematic Review. Materials.

[16] Abad-Coronel C., Palomeque A., Mena Córdova N., Aliaga P. (2022). Digital volumetric analysis of CAD/CAM polymeric materials after tooth brushing. Polymers.

[17] Meseli S., Alkan E., Korkut B., Kanar O., Tagtekin D. (2025). Abrasiveness and bleaching level of toothpastes on composite resins: A quantitative analysis using a novel brushing simulator. Appl. Sci..

[18] Al Khuraif A.A.A. (2014). An in vitro evaluation of wear and surface roughness of particulate filler composite resin after tooth brushing. Acta Odontol. Scand..

[19] Ulku S.G., Unlu N. (2024). Factors influencing the longevity of posterior composite restorations: A dental university clinic study. Heliyon.

[20] Shekhar S., Suprabha B.S., Shenoy R., Natarajan S., Rao A. (2022). Comparative evaluation of surface roughness and wettability of an alkasite with nano bulk-fill and nanofilled resin composite restorative materials: An in vitro study. Contemp. Clin. Dent..

[21] Mehl A., Koch R., Zaruba M., Ender A. (2013). 3D monitoring and quality control using intraoral optical camera systems. Int. J. Comput. Dent..

[22] Dettwiler C., Meller C., Eggmann F., Saccardin F., Kühl S., Filippi A., Krastl G., Weiger R., Connert T. (2018). Evaluation of a fluorescence-aided identification technique (FIT) for removal of composite bonded trauma splints. Dent. Traumatol..

[23] Ender A., Attin T., Mehl A. (2016). In vivo precision of conventional and digital methods of obtaining complete-arch dental impressions. J. Prosthet. Dent..

[24] Zaruba M., Ender A., Mehl A. (2014). New applications for three-dimensional follow-up and quality control using optical impression systems and OraCheck. Int. J. Comput. Dent..

[25] Mehl A., Gloger W., Kunzelmann K.H., Hickel R. (1997). A new optical 3-D device for the detection of wear. J. Dent. Res..

[26] Klein C., von Ohle C., Wolff D., Meller C. (2022). A quantitative assessment of silicone and PTFE-based stamp techniques for restoring occlusal anatomy using resin-based composites. Clin. Oral Investig..

[27] Cohen J. (2013). Statistical Power Analysis for the Behavioral Sciences.

[28] Pradhan D., Parihar V., Tiwari S., Parihar S., Mulchandani A. (2025). Occlusal stamp technique: A biomimetic approach in crafting occlusal topography. World J. Dent..

[29] Balci M., Sabah G.A., Kanmaz M.G., Eyici G., Ayan K., Saklakoğlu N. (2026). In Vitro Comparison of Four Resin Composite Matrix Systems. Oper. Dent..

[30] Zhu J., Fu C., Deng X., Ma L., Song F., Huang C. (2024). Effects of stamp material and restoration depth on the accuracy of direct composite resin restorations using stamp technique. J. Dent..

[31] Sozen Yanik I., Sahin Hazir D., Aktas G., Guncu M.B. (2026). Do CAD/CAM restorative materials respond differently to coffee thermocycling and simulated toothbrushing?. Clin. Oral Investig..

[32] Dindar M.B., Atay M.T. (2023). The effect of brushing force on the surface properties and color stability of dental enamel. Necmettin Erbakan Üniv. Diş Hekim. Derg..

[33] (2007). Dental Materials—Guidance on Testing of Wear—Part 1: Wear by Toothbrushing.

[34] Basheer N., Madhubala M.M., Mahalaxmi S. (2020). Future perspectives of biomimetics in restorative dentistry. J. Pharm. Res. Int..

[35] Murdoch-Kinch C.A., McLean M.E. (2003). Minimally invasive dentistry. J. Am. Dent. Assoc..

[36] Bal H., Yazıcıoğlu O. (2025). Treatment of Teeth with Intact Occlusal Morphology Using the Stamp Technique with Different Impression Materials: Case Report. Selcuk Dent. J..

[37] Zotti F., Vincenzi S., Zangani A., Bernardi P., Sbarbati A. (2023). Stamp technique: An explorative SEM analysis. Dent. J..

[38] Lee I., Chang J., Ferracane J. (2008). Slumping resistance and viscoelasticity prior to setting of dental composites. Dent. Mater..

[39] Al-Ahdal K., Silikas N., Watts D.C. (2014). Rheological properties of resin composites according to variations in composition and temperature. Dent. Mater..

[40] Baroudi K., Mahmoud S. (2015). Improving composite resin performance through decreasing its viscosity by different methods. Open Dent. J..

[41] Darabi F., Tayefeh-Davalloo R., Tavangar S.M., Naser-Alavi F., Boorboo-Shirazi M. (2020). The effect of composite resin preheating on marginal adaptation of class II restorations. J. Clin. Exp. Dent..

[42] Dionysopoulos D., Papadopoulos C., Koliniotou-Koumpia E. (2014). The evaluation of various restoration techniques on internal adaptation of composites in class v cavities. Int. J. Biomater..

[43] Baltacioğlu İ.H., Demirel G., Öztürk B., Aydin F., Orhan K. (2024). Marginal adaptation of bulk-fill resin composites with different viscosities in class II restorations: A micro-CT evaluation. BMC Oral Health.

[44] Scepanovic D., Par M., Attin T., Tauböck T.T. (2022). Marginal Adaptation of Flowable vs Sonically Activated or Preheated Resin Composites in Cervical Lesions. J. Adhes. Dent..

[45] Lee I.B., Son H.H., Um C.M. (2003). Rheologic properties of flowable, conventional hybrid, and condensable composite resins. Dent. Mater..

[46] Assiri A., Alomairy A., Magdy N.M. (2018). Marginal adaptation of bulk-fill versus layered resin composite restorations. Int. J. Health Sci. Res..

[47] Soares B.M., Barbosa M.P., de Almeida R.V., Jardim R.N., da Silva E.M. (2024). Marginal integrity and physicomechanical properties of a thermoviscous and regular bulk-fill resin composites. Clin. Oral Investig..

[48] Kantovitz K., Cabral L., Carlos N., de Freitas A., Peruzzo D., Franca F., do Amaral F., Basting R., Puppin-Rontani R. (2021). Impact of resin composite viscosity and fill-technique on internal gap in class I restorations: An OCT evaluation. Oper. Dent..

[49] Shahidi C., Krejci I., Dietschi D. (2017). In Vitro Evaluation of Marginal Adaptation of Direct Class II Composite Restorations Made of Different “Low-Shrinkage” Systems. Oper. Dent..

[50] Andrade A.C.M., Trennepohl A.A., Moecke S.E., Borges A.B., Torres C.R.G. (2022). Viscosity modulation of resin composites versus hand application on internal adaptation of restorations. Clin. Oral Investig..

[51] Shibasaki S., Takamizawa T., Nojiri K., Imai A., Tsujimoto A., Endo H., Suzuki S., Suda S., Barkmeier W., Latta M. (2017). Polymerization behavior and mechanical properties of high-viscosity bulk fill and low shrinkage resin composites. Oper. Dent..

[52] GC Corporation (2018). G-ænial Universal Injectable Technical Manual.

[53] Han S.-H., Lee I.-B. (2018). Effect of vibration on adaptation of dental composites in simulated tooth cavities. Korea-Aust. Rheol. J..

[54] Yang H., Xu C., He L., Tian J. (2025). Wear resistance of three direct resin composites in artificial saliva at varying pH levels. Front. Dent. Med..

[55] Alkhudhairy F. (2017). Wear resistance of bulk-fill composite resin restorative materials polymerized under different curing intensities. J. Contemp. Dent. Pract..

[56] Shimokawa C.A.K., Giannini M., André C.B., Sahadi B.O., Faraoni J.J., Palma-Dibb R.G., Soares C., Price R. (2019). In vitro evaluation of surface properties and wear resistance of conventional and bulk-fill resin-based composites after brushing with a dentifrice. Oper. Dent..

[57] Osiewicz M.A., Werner A., Roeters F.J., Kleverlaan C.J. (2022). Wear of bulk-fill resin composites. Dent. Mater..

[58] Asadian F., Hoseini A.P., Ahmadian L., Rafeie N., Rezaei S., Moradi Z. (2022). In vitro attrition wear resistance of four types of paste-like bulk-fill composite resins. BMC Oral Health.

[59] Elsahn N.A., El-Damanhoury H.M., Shirazi Z., Saleh A.R.M. (2023). Surface Properties and Wear Resistance of Injectable and Computer-Aided Design/Computer Aided Manufacturing–Milled Resin Composite Thin Occlusal Veneers. Eur. J. Dent..

[60] Ozdemir S.B., Ozdemir B. (2026). Wear resistance, microhardness and compressive strength of high filled flowable composite resins. Sci. Rep..

[61] Bayne S.C. (2005). Dental biomaterials: Where are we and where are we going?. J. Dent. Educ..

[62] Ferracane J.L. (2011). Resin composite--state of the art. Dent. Mater..

[63] Gkantidis N., Dritsas K., Ren Y., Halazonetis D., Katsaros C. (2020). An accurate and efficient method for occlusal tooth wear assessment using 3D digital dental models. Sci. Rep..

[64] Mourouzis P. (2025). Critical methodological factors influencing the accuracy of intraoral scanners in digital dentistry research. Comput. Biol. Med..

[65] Park K., Limpuangthip N., Kim S.J., Yeo I.S.L., Lee J.H., Srinivasan M. (2025). Clinical accuracy and responsiveness of 3D software for measuring facial dimensions at altered vertical dimensions. J. Dent..

[66] Korkut B., Şenol A.A., Saygılı C.C., Kaya B.D., Gresnigt M., Özcan M. (2026). Clinical assessment of scanning deviations of four intraoral scanner systems following the cut-out and rescan procedures with dental dam isolation. J. Prosthet. Dent..

